# Highly sensitive strain sensor based on helical structure combined with Mach-Zehnder interferometer in multicore fiber

**DOI:** 10.1038/srep46633

**Published:** 2017-04-18

**Authors:** Hailiang Zhang, Zhifang Wu, Perry Ping Shum, Xuan Quyen Dinh, Chun Wah Low, Zhilin Xu, Ruoxu Wang, Xuguang Shao, Songnian Fu, Weijun Tong, Ming Tang

**Affiliations:** 1 CINTRA CNRS/NTU/Thales, UMI 3288, 50 Nanyang Drive, 637553 Singapore; 2 COFT, School of EEE, Nanyang Technological University, 50 Nanyang Avenue, 639798 Singapore; 3 Thales Solutions Asia Pte Ltd, R&T, 28 Changi North Rise, 498755 Singapore; 4 National Engineering Laboratory for Next Generation Internet Access System, School of Optical and Electronic Information, Huazhong University of Science and Technology, Wuhan 430074, China; 5 Yangtze Optical Fibre and Cable Company Ltd (YOFC), 4# Guanshan Er Road, Wuhan 430073, China

## Abstract

Optical fiber sensors for strain measurement have been playing important roles in structural health monitoring for buildings, tunnels, pipelines, aircrafts, and so on. A highly sensitive strain sensor based on helical structures (HSs) assisted Mach-Zehnder interference in an all-solid heterogeneous multicore fiber (MCF) is proposed and experimentally demonstrated. Due to the HSs, a maximum strain sensitivity as high as −61.8 pm/με was experimentally achieved. This is the highest sensitivity among interferometer-based strain sensors reported so far, to the best of our knowledge. Moreover, the proposed sensor has the ability to discriminate axial strain and temperature, and offers several advantages such as repeatability of fabrication, robust structure and compact size, which further benefits its practical sensing applications.

Optical fiber sensors have attracted considerable attention due to the advantages such as compactness, light weight, high stability, repeatability, and immunity to electromagnetic interference. Strain measurement, one of the most important applications of optical fiber sensors, has been widely applied in many fields, especially in structural health monitoring for aircrafts, dams, towers, bridges, skyscrapers, railways, highways, and so on. In the past few years, fiber-based strain sensors have been demonstrated with various types of schemes, for example, a fiber Bragg grating (FBG) in a waveguide-array microstructured optical fiber^1^ or an all-solid photonic bandgap fiber^2^, a long-period fiber grating (LPFG) in an index-guiding photonic crystal fiber (PCF)^3^ or a multicore fiber (MCF)^4^, in-line Mach-Zehnder interferometers (MZIs)^2^,^5^,^6^,^7^ and all-fiber Fabry-Pérot interferometers (FPIs)^8^,^9^,^10^,^11^. Their sensitivities to strain were typically about 1 pm/με for FBGs, less than 10 pm/με for LPFGs or MZIs, and less than 16 pm/με for FPIs. In order to further enhance the strain sensitivity, some additional designs have been demonstrated based on FPIs. For instance, a FPI based on a rectangular-shape air cavity with the wall thickness of about 1 μm achieved a strain sensitivity as high as 43.0 pm/με^12^. By combining Vernier effect, another FPI consisting of two cascaded air cavities was reported to achieve a strain sensitivity of 47.14 pm/με^13^. However, because of the air-cavities of FPI structures, the devices have a low mechanical strength^8^. The previously reported sensing schemes encounter the problems of a relatively low sensitivity or weak mechanical strength. Therefore, strain sensors with a high sensitivity and a robust mechanical structure are in high demand.

In this paper, we will demonstrate another approach to achieve ultra-high strain sensitivity by introducing helical structures (HSs) into a MCF. HSs in optical fibers, also called chiral structures, refer to the fiber structures that are deformed into spring shapes rather than conventional fibers with straight cores. This special structure is usually fabricated by continuously twisting optical fibers when they pass through a miniature heat zone^14^,^15^. Since V. I. Kopp *et al*. reported two types of chiral LPFGs by twisting an optical fiber with a noncircular core into HSs in 2004^15^, such special fiber structure has attracted increasing interest due to its unique polarization dependence as well as very promising sensing applications^16^,^17^. For example, V. I. Kopp’s group demonstrated sensors for measurement of liquid level and temperature^16^, pressure and temperature^18^. Besides, P. St. J. Russell’s group demonstrated helical PCFs to convert the fundamental core mode to a series of cladding orbital angular momentum (OAM) states at certain wavelengths, causing a few dips in the transmission spectrum^19^. Afterwards, they reported a sensor for simultaneous measurement of axial strain and twist by utilizing a helical PCF, the strain sensitivity was about 1.18 pm/με^20^.

In our work, an all-solid heterogeneous MCF was locally twisted into helical structures and then spliced between two short sections of multimode fibers (MMFs) to construct an in-line MZI. The HSs were fabricated in the MCF by a CO_2_ laser splicing system. In the region of the HSs, the outer cores were deformed into spring-like structures while the center core was kept straight. Due to the HSs, a maximum strain sensitivity as high as −61.8 pm/με was achieved. It is about 56 times higher than that of the same MCF-based Mach-Zehnder interferometer^5^. Since the sensor is composed of all-solid fibers which have the same cladding diameter, it has a relatively better mechanical strength compared with air-cavity-based schemes. Furthermore, the proposed sensor can also measure strain and temperature simultaneously. The cross sensitivity to temperature can be eliminated.

## Results and Discussion

**Figure 1:**
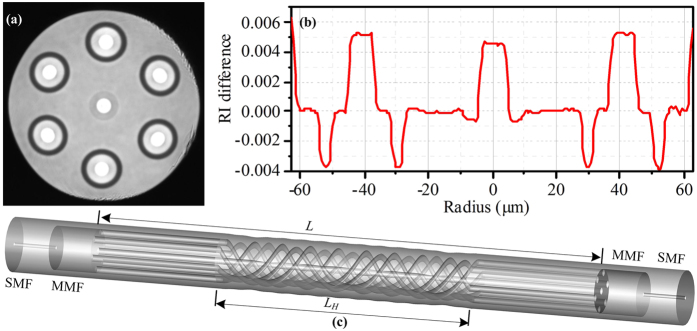
(**a**) Microscope image of the cross section of the MCF. (**b**) Refractive index profile of the MCF. (**c**) The configuration of the proposed sensor.

The microscope image of the cross section of the MCF used in this work is shown in Fig. 1(a). The MCF consists of six identical outer cores and one center core. The cladding diameter, core pitch and core diameter are 125 μm, 42 μm and 8.4 μm, respectively. The six outer cores are designed with G.657.B3 refractive index (RI) profile, and they have deep trenches. The center core is designed with G.652 RI profile and a shallow trench. Figure 1(b) illustrates the RI profile of the MCF. Compared with the pure silica cladding, the RI differences of the outer cores, center core, trenches of outer cores and trench of center core are about 5.3 × 10^−3^, 4.7 × 10^−3^, −3.8 × 10^−3^ and −6 × 10^−4^, respectively. More geometrical information about the MCF can be found in our previous paper^21^. Figure 1(c) illustrates the schematic diagram of the proposed in-line MZI-based sensor structure, in which a segment of MCF with HSs is spliced between two segments of MMFs, and then connected to a light source and an optical spectrum analyzer (OSA) with single mode fibers (SMFs). One of the MMFs is utilized to couple the light into all the seven cores and the cladding of the MCF, while the other MMF is used to recombine the light into the lead-out SMF. It should be noted that all the fusion splicing is implemented without lateral offset. The cladding diameter and core diameter of the MMFs are 125 μm and 105 μm, respectively. The lengths of the two MMFs should be chosen to be as short as possible, so that the phase differences of their guided modes could be neglected^22^. When the MMF length was 1 mm, almost no interference was generated by itself (see Supplementary Fig. S1). Even though the MMF length can affect the coupling efficiency, larger or smaller insertion loss caused by MMFs will not obviously affect the proposed sensor performance. The simulation of investigating the influence of the MMF length on the coupling efficiency can be found in the second paragraph in Supplementary Information (see Supplementary Fig. S2).

**Figure 2:**
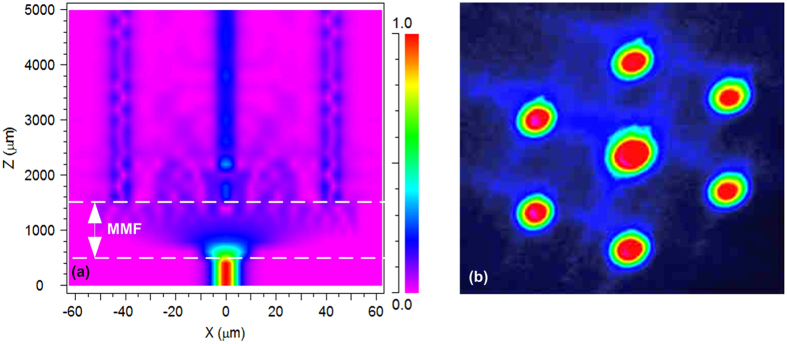
(**a**) Simulated light propagation along the SMF-MMF-MCF(without HSs) structure when the MMF length was 1 mm. (**b**) Measured light distribution at the end facet of the MCF without HSs.

To demonstrate that the light can be coupled into all the seven cores of the MCF within the proposed sensor, beam propagation method (BPM) was utilized to simulate the light propagation in a SMF-MMF-MCF structure. It’s worth noting that, to simplify the simulation, the MCF section was set to be without HSs. In the simulation, the refractive indices were set to be 1.4607/1.444, 1.449/1.444 for the core /cladding of the MMF and SMF, respectively. The core diameter and length of the SMF were set to be 8.2 μm and 500 μm. The lengths of the MMF and MCF were set to be 1000 μm and 3500 μm, respectively. Figure 2(a) shows the simulated electric field intensity distribution along the SMF-MMF-MCF structure at the wavelength of 1550 nm when the MMF length was 1 mm. As can be seen, the incident light from the SMF expanded obviously in the MMF section and then was coupled into the center core, outer cores and cladding of the MCF. Furthermore, the near-field light distribution at the end facet of the MCF without HSs was measured by utilizing a microscope objective lens and a CCD camera at 1550 nm. As shown in Fig. 2(b), all the seven cores were coupled with light obviously and a proportion of light was distributed in the cladding as well. The light in the center core was stronger than that in the outer cores. The measured result is accordance with the simulation result.

For our proposed sensor, the length of the twisted part is about 750 μm (only very few periods), which are too few to generate apparent LPFG effect compared with the chiral LPFG reported by V. I. Kopp^17^. On the other hand, the RI differences among the center core, outer cores and cladding, and the length difference between the center core and the outer cores induced by the HSs, will result in phase differences among the modes propagating along the MCF. Thus, a series of Mach-Zehnder interferences can occur among the outer cores mode, center core mode and cladding modes. Hence, we will focus on the operation principle of our proposed sensor configuration by analyzing the interferences in it. For an interference pattern generated by *N* different compositions, the intensity can be expressed as:


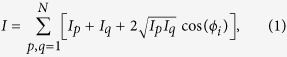


where *p* ≠ *q, I*_*p*_ and *I*_*q*_ represent the intensities of *p*-th mode and *q*-th mode. *ϕi* denotes the accumulated phase difference between *p*-th mode and *q*-th mode. Despite the fact that more than one cladding modes may be excited and propagate through the MCF, it can be assumed that only one cladding mode dominates the interference with the core modes^23^,^24^. In addition, since all the six outer cores have the same specifications, the light in the six outer cores is regarded as a whole to be involved in the interferences.

When the light passes through the sensor, the accumulated phase differences between the center core mode and cladding mode, the center core mode and outer core mode, the outer core mode and cladding mode, can be expressed by the following three formulas, respectively:


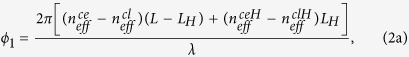



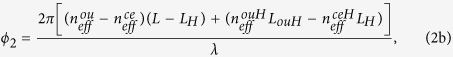



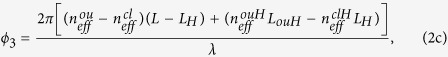


where 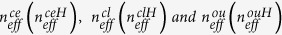 represent the refractive indices of the center core mode, the cladding mode and the outer core mode in the non-helical part (helical part), respectively. *L, L*_*H*_ and *L*_*ouH*_ represent the lengths of the total MCF, the center core and outer cores in the helical part, respectively. *λ* is the operating wavelength. The length of the helical part of the outer cores can be calculated by *L*_*ouH*_ = [(2*πd*)^2^ + [2*πL*_*H*_/*φ*]^2^]^1/2^*φ*/2*π*, where *d* is the distance between the outer core and the center core, *φ* represents the total torsion angle of the helical structure.

When the phase difference *ϕ* = (2*m* + 1)*π (m* = 0, 1, 2, 3…), the destructive interference condition is satisfied. By substituting this condition into Eq. (2a), Eq. (2b) and Eq. (2c), the respective resonant wavelength dips can be written as:


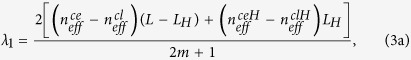



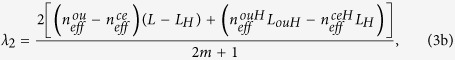



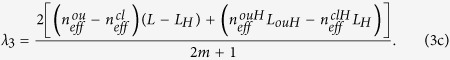


when external axial strain *ε* is applied to the sensor, the dimension of the sensor and the refractive indices of the propagating modes will be changed. *ε* is defined as the relative length change of the fiber, i.e. *ε* = Δ*L/L* = Δ*L*_*H*_/*L*_*H*_, where Δ*L* and Δ*L*_*H*_ represent the strain-induced length changes of the whole MCF and the center core in helical part, respectively. The radial strain *ε*_*r*_ = −*vε, v* is the Poisson’s ratio with a typical value of 0.16 for optical fibers^25^. Hence, the distance between the outer core and the center core under the externa axial strain *ε* can be expressed as *d*_*ε*_ = (1 − *vε)d*.

According to Eq. (3a), the strain-induced wavelength shift for *λ*_1_ can be approximately expressed as^26^,^27^:


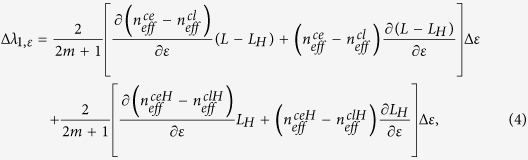


where Δ*ε* represents the axial strain variation. Since ∂(*L* − *L*_*H*_)/∂*ε* and ∂*L*_*H*_/∂*ε* can be approximated as (*L* − *L*_*H*_) and *L*_*H*_, respectively, *n*_*eff*_^*ce*^ ≈ *n*_*eff*_^*ceH*^, *n*_*eff*_^*cl*^ ≈ *n*_*eff*_^*clH*^, and *L* is much larger than *L*_*H*_, Eq. (4) can be simplified as:


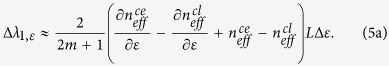


∂*n*_*eff*_^*ce*^/∂*ε* and ∂*n*_*eff*_^*cl*^/∂*ε* are determined by the effective photo-elastic coefficients, and both of them do not vary with the axial strain. Therefore, according to Eq. (5a), we can see that Δλ_1,*ε*_ is proportional to axial strain, the corresponding wavelength dip will shift linearly when the axial strain increases.

Similarly, the strain-induced wavelength shifts for *λ*_2_ and *λ*_3_ can be approximately described by:


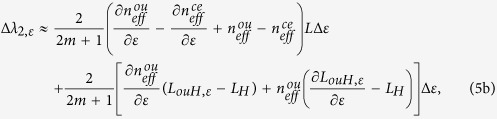



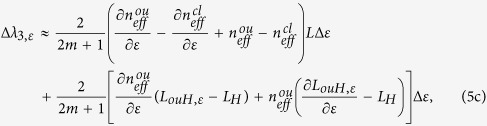


where *L*_*ouH,ε*_ = [(*φd*_*ε*_)^2^ + [*L*_*H*_(1 + *ε*)]^2^]^1/2^. For both Eq. (5b) and Eq. (5c), the first terms change linearly while the second terms change nonlinearly with axial strain variation. Therefore, both *λ*_2_ and *λ*_3_ will shift nonlinearly to axial strain variation.

To demonstrate the HSs can improve the strain sensitivity, we analyze Eq. (5b) for the two cases, i.e. *φ* = 0 and *φ* > 0. If *φ* = 0, which means no HSs are involved in the MCF, *L*_*ouH,ε*_ = *L*_*H*_, and ∂*L*_*ouH,ε*_/∂*ε* = *L*_*H*_. Thus, Eq. (5b) can be simplified as:


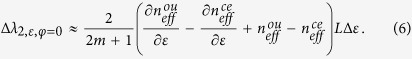


If *φ* > 0, *L*_*ouH,ε*_ > *L*_*H*_ and ∂*L*_*ouH,ε*_/∂*ε* < *L*_*H*_. In addition, 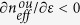 and 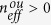, thus


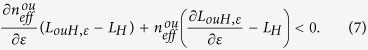


Since Δ*λ*_2,ε,*ϕ* = 0_ < 0, when *φ* > 0, according to Eq. (5b) and inequality (7), |Δ*λ*_2,ε,*ϕ* = 0_| < |Δ*λ*_2,*ε*|_, which shows that the corresponding wavelength dip can have a larger wavelength shift when fabricating HSs in the MCF. In other words, the HSs in the MCF can improve the strain sensitivity.

**Figure 3:**
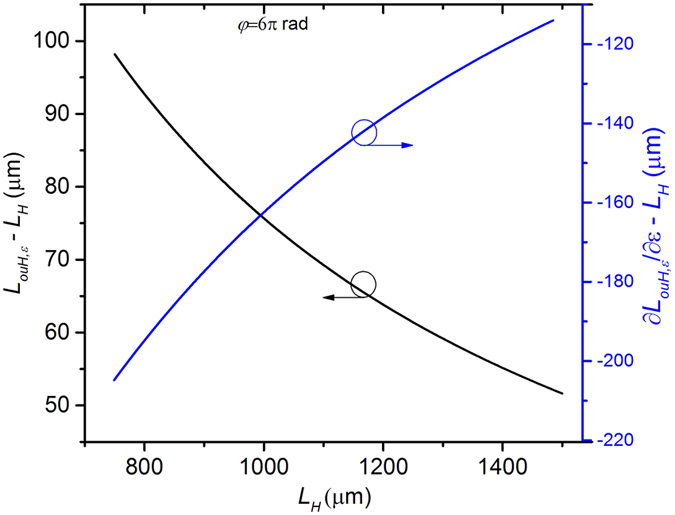
Variations of (*L*_*ouH,ε*_ − *L*_*H*_) and (∂*L*_*ouH,ε*_/∂*ε* − *L*_*H*_) with increasing *L*_*H*_ when *φ* = 6*π* rad.

To investigate the influence of *L*_*H*_ on the strain sensitivity, the variations of (*L*_*ouH,ε*_ − *L*_*H*_) and (∂*L*_*ouH,ε*_/∂*ε* − *L*_*H*_) with increasing *L*_*H*_ for a certain strain (e.g. *ε* = 200 με), were calculated, the results are shown in Fig. 3. For a certain total torsion angle, e.g. *φ* = 6π rad, (*L*_*ouH,ε*_ − *L*_*H*_) decreases and (∂*L*_*ouH,ε*_/∂*ε* − *L*_*H*_) increases nonlinearly with increasing *L*_*H*_, as shown in Fig. 3. These variation trends result in |Δ*λ*_2,*ε*_| decreasing. In other words, for a certain *φ*, the strain sensitivity becomes smaller with increasing *L*_*H*_. When the applied strain increases, *L*_*H*_ is stretched to be longer, thus the strain sensitivity decreases. This reveals that the wavelength dips generated by the outer core mode interfering with the cladding mode or the center core mode have nonlinear strain responses.

When temperature is changed, both the RI and dimension of the sensing fiber will be changed. Since the thermal-optic coefficient (8.6 × 10^−6^/°C) is much larger than the thermal expansion coefficient (5.5 × 10^−7^/°C) for silica^28^, the dimension change induced by temperature variation can be neglected. Therefore, the wavelength shifts of the three resonant dips due to the temperature variation Δ*T* can be described by the following formulas, respectively:


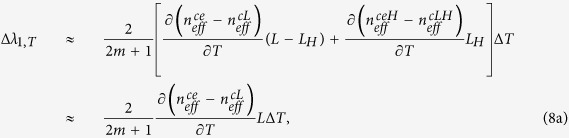



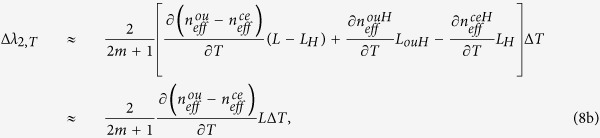



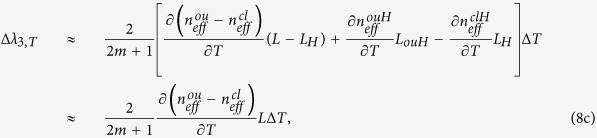


where *T* represents the temperature. According to Eq. (8a), Eq. (8b) and Eq. (8c), all the dips will shift linearly to longer wavelengths with temperature increasing. The thermal-optic coefficient of the pure silica cladding is smaller than that of the germanium-doped silica cores^29^. In addition, the thermal-optic coefficients of the center core and outer cores are similar, although there is a slight difference on the doping concentrations between the center core and outer cores. Therefore, the difference of the thermal-optic coefficients between the center core and outer cores is smaller than that between the cladding and cores. In other words, ∂(*n*_*eff*_^*ou*^ − *n*_*eff*_^*ce*^)/∂*T* is smaller than ∂(*n*_*eff*_^*ce*^ − *n*_*eff*_^*cl*^)/∂*T* and ∂(*n*_*eff*_^*ou*^ − *n*_*eff*_^*cl*^)/∂*T*, which results in that the resonant dip generated from the interference between the center core mode and outer core mode has a smaller temperature sensitivity than the other two types of resonant dips.

**Figure 4:**
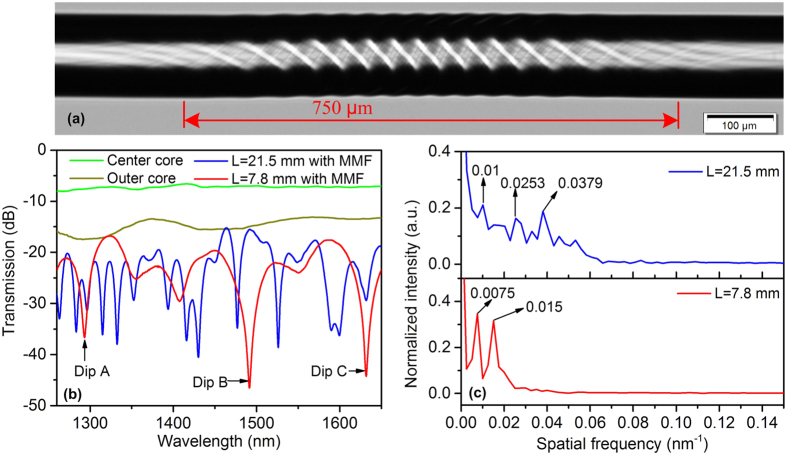
(**a**) Side view microscope image of the MCF with HSs. (**b**) Transmission spectra of the MCF with HSs for different configurations. (**c**) Spatial frequency spectra of the corresponding transmission spectra.

Figure 4(a) illustrates the microscope image of the side view of the HSs with a total pre-torsion of 6π rad. The length of the twisted region is approximately 750 μm. To measure the transmission spectra of different cores of the MCF, the sample with a total length of about 20 mm was spliced between a pair of fan-in/out 1 × 7 multiplexers^5^. The total insertion loss of the two fan-in/out multiplexers is about 5 dB. The green curve and dark yellow curve in Fig. 4(b) represent the measured transmission spectra of the center core and one outer core, respectively. As shown, no obvious dips were observed in the spectra for both the center core and outer core. The spectrum of the outer core had a lager loss, which was mainly caused by the lager structure deformation and relatively larger mode-field mismatch with the multiplexers. The transmission spectra were measured again when the MCF with HSs was spliced between two segments of MMFs and then spliced between two SMFs. The lengths of the MMFs were about 1 mm. The total insertion loss of the devices was about 17 dB. The blue curve and red curve in Fig. 4(b) represent the transmission spectra when the lengths of MCF were about 21.5 mm and 7.8 mm, respectively. As shown, obvious inhomogeneous interferences spectra were obtained. This result suggests that the interference patterns were superposition of multiple interferences with different free spectrum ranges (FSRs). This is further validated by the corresponding spatial frequency spectra obtained by taking fast Fourier Transform (FFT), as shown in Fig. 4(c).

For a normal interferometer, e.g., if the MCF is not twisted, the relationship between the differential modal group index and spatial frequency can be described by^30^:


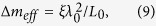


where Δ*m*_*eff*_, *ξ, λ*_0_ and *L*_0_ represent the differential modal group index, the spatial frequency, the center wavelength and the interferometer length, respectively. For our proposed sensor structure, since the length of the twisted region was much smaller than the total length of the MCF, we can still use Eq. (9) to roughly analyze the spatial frequency. When the length of MCF was 21.5 mm, there were three strong peaks and a few weaker peaks in the spatial frequency spectrum. The three strongest peaks locate at 0.01 nm^−1^, 0.0253 nm^−1^ and 0.0379 nm^−1^. The Δ*m*_*eff*_ corresponding to peak 0.01 nm^−1^ was approximately calculated to be 9.8 × 10^−4^, which is close to the material RI difference between the center core and the outer cores. For 0.0253 nm^−1^ and 0.0379 nm^−1^, the corresponding Δ*m*_*eff*_ were calculated to be 2.8 × 10^−3^ and 3.7 × 10^−3^. Moreover, the order of magnitude of the material RI difference between the center core and cladding, as well as that between the outer cores and cladding is minus three. Hence the inhomogeneous interference pattern should be mainly caused by the superposition of the interferences between the center core mode and outer core mode, center core mode and cladding mode, outer core mode and cladding mode. The weaker peaks in the spatial frequency spectrum could be attributed to non-uniformity of the outer cores and the excited high-order cladding modes. When the length of the MCF was 7.8 mm, the spatial frequency spectrum only had two dominant peaks locating at 0.0075 nm^−1^ and 0.015 nm^−1^. Their corresponding Δ*m*_*eff*_ were calculated to be 2.0 × 10^−3^ and 4.0 × 10^−3^. It implies that the total interference pattern contained at least the interference between the center core mode and cladding mode and that between the outer core mode and cladding mode. The spatial frequency component of the interference between the center core mode and outer core mode was missing in the FFT spectrum due to the limit range of the measured optical spectrum shown in Fig. 4(b) and its much larger FSR. The FSR of the interference between the center core mode and outer core mode was around 450 nm, which could be estimated by FSR ≈ *λ*_0_^2^/(Δ*m*_*eff*_*L*). Nevertheless, it is still possible that one of the resonant dips shown in Fig. 4(b) with red curve was generated from the interference between the center core mode and outer core mode. The resonant dips originated from different interferences are expected to respond differently to strain, temperature, or other parameters.

**Figure 5:**
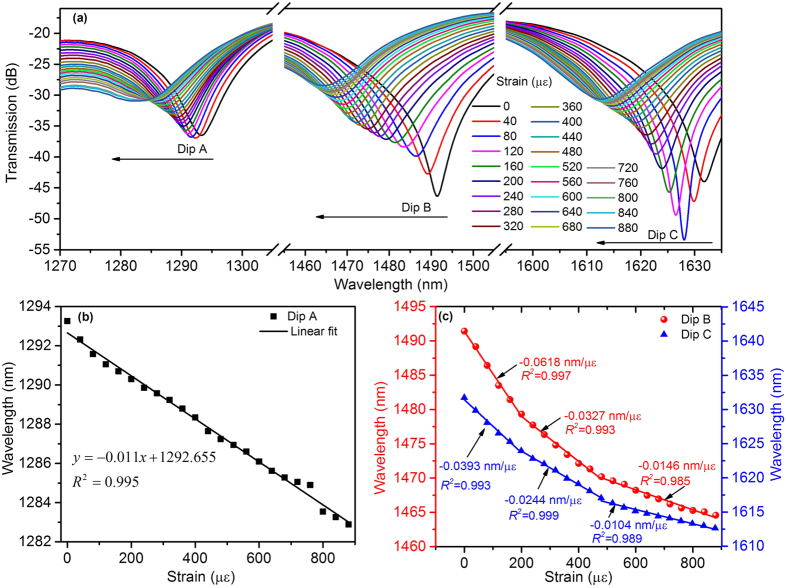
(**a**) Spectral shift of Dip A, Dip B and Dip C with varying axial strain. (**b**) Wavelength response of Dip A to axial strain. (**c**) Wavelength responses of Dip B and Dip C to axial strain.

The performance of the sample with the MCF length of 7.8 mm was characterized as follows. When the axial strain was increased from 0 to 880 με, Dip A, Dip B and Dip C shifted to shorter wavelengths, as illustrated in Fig. 5(a). The center wavelength of Dip A responded to axial strain linearly and the corresponding sensitivity was measured to be −0.011 nm/με with the coefficient of determination (R-square) of about 0.995, as shown in Fig. 5(b). Different from Dip A, both Dip B and Dip C shifted to shorter wavelengths nonlinearly with different strain sensitivities. By piecewise linearly fitting the measured wavelengths, the linear strain sensitivities of Dip B and Dip C were −0.0618 nm/με and −0.0393 nm/με in the strain range from 0 to 200 με, −0.0327 nm/με and −0.0244 nm/με in the strain range from 200 to 480 με, −0.0146 nm/με and −0.0104 nm/με in the strain range from 480 to 880 με, respectively, as shown in Fig. 5(c). The strain responses of Dip B and Dip C were nonlinear for whole strain range from 0 to 880 με, and their strain sensitivities were much smaller in larger strain range. According to the aforementioned theoretical analysis on the strain responses, Dip A should be generated from the interference between the center core mode and cladding mode because of its linear response to the axial strain, while Dip B and Dip C should be dominated by the outer core mode interfering with the cladding mode or the center core mode.

**Figure 6:**
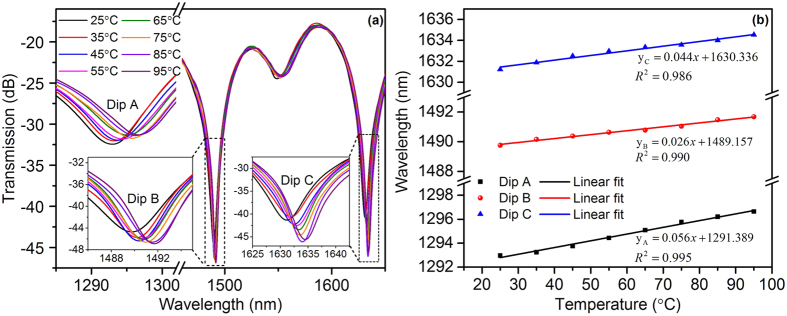
(**a**) Transmission spectra of the proposed sensor with varying temperature. The insets show the spectral shifts of Dip B and Dip C. (**b**) Wavelength responses of Dip A, Dip B and Dip C to temperature variation.

As shown in Fig. 6(a), all the three dips shifted to longer wavelengths when the sensor was heated from 25 °C to 95 °C with a step of 10 °C. The corresponding temperature sensitivities of Dip A, Dip B and Dip C, as show in Fig. 6(b), were measured to be 0.056 nm/°C, 0.026 nm/°C and 0.044 nm/°C, respectively. Comparing with Dip A and Dip C, Dip B has a much smaller temperature sensitivity. Since the difference of the thermal-optic coefficients between the center core and outer cores is smaller than that between the cladding and cores, the resonant dip generated from the interference between the center core mode and outer core mode has a smaller temperature sensitivity than the resonant dips generated from the interference between the cladding mode and center core mode as well as that between the cladding mode and outer core mode. Therefore, Dip B should be generated from the interference between the outer core mode and the center core mode. Combining all the aforementioned discussion, we can infer that Dip C should be dominated by the interference between the outer core mode and cladding mode. The experimental results of the characterizations to axial strain and temperature match the theoretical prediction very well.

Moreover, since the three dips response differently to axial strain and temperature, by selecting two dips as the sensing indicators, e.g., Dip A and Dip B, the proposed sensor can be utilized to measure axial strain and temperature simultaneously by constructing matrixes^1^:


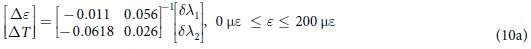



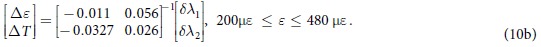



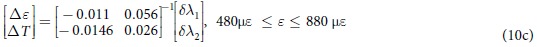


## Conclusion

We have proposed and experimentally demonstrated a highly sensitive strain sensor with a compact size based on helical structures fabricated in an all-solid heterogeneous multicore fiber. The length of the sensor head is about 1 cm. The proposed sensor exhibits a high strain sensitivity of −61.8 pm/με in the strain range from 0 to 200 με. To the best of our knowledge, this is the highest strain sensitivity comparing with the previously reported strain sensors based on all-fiber interferometers. Furthermore, this sensor can be applied to discriminate axial strain and temperature. Besides, this device has a relatively better mechanical strength because all the segments are all-solid fibers with the same physical diameter. With the advantages such as low cost, repeatability of fabrication, compact size, robust structure, high sensitivity, and strain-temperature discrimination, the proposed strain sensor shows great potential in the applications of structural health monitoring.

## Methods

### Helical structure fabrication

**Figure 7:**
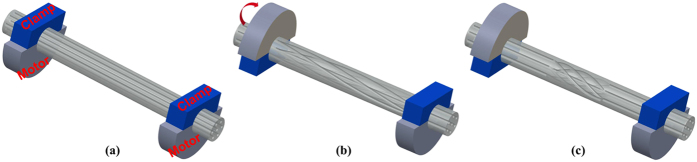
Schematic diagrams of the process of fabricating the helical structures. (**a**) Before adding pre-torsion. (**b**) The pre-torsion is distributed along the MCF without laser heating. (**c**) The pre-torsion is concentrated into a small region after heating.

The HSs were fabricated by using the CO_2_ laser splicing system (Fujikura, LZM-100) with manual controlling mode. The total twisted angle of the HSs for the proposed sensor sample used in this paper is 6 π rad. Figure 7 illustrates the process of fabricating the HSs in the MCF. It involves three main steps. In step 1, as shown in Fig. 7(a), a segment of the MCF with the coating layer stripped was fixed straightly between the two stages of LZM-100. The distance between the two stages was about 45 mm. In step 2, as shown in Fig. 7(b), the rotation motor of one stage was rotated by 1 π. Then the 1 π pre-torsion was distributed along the whole MCF between the two stages. In step 3, as shown in Fig. 7(c), after using the CO_2_ laser to heat the MCF, the pre-torsion of 1 π was concentrated into a small region. Since the tuning range of the torsion for the two rotation motors is from −1 π to 1 π, in order to apply larger pre-torsion into the MCF, the rotation motor of the other stage was rotated by −1 π, then the MCF was heated by the laser. Thus a pre-torsion of 2 π was introduced into the small region of the MCF. Then the angles of two rotation motors were set to be 0°, and the two theta motors rotated automatically to their original position along the same direction. The aforementioned steps were repeated until a total pre-torsion of 6π was applied into the MCF. Owing to the programmable controller and the stability of LZM-100, the HSs fabrication is repeatable and simple. Furthermore, the twisted angle can also be controlled flexibly.

### Characterization method

An OSA (Yokogawa AQ6370c) and a stabilized broadband light source (Infinon Research) were used to observe the transmission spectra. In order to investigate the strain response, the proposed sensor was clamped straightly between two stages with a distance of about 250 mm. One of the stages was fixed, while the other one was moved axially to the opposite direction to stretch the sensor. As the cross-sectional area and Young’s Modulus of the MCF are almost the same with those of the MMFs and SMFs, the axial strain *ε* applied on the sensor can be calculated by the relative length change of the fiber. Temperature responses of the three dips were characterized by putting the fiber with the sensor head in the groove of a column oven with temperature precision of 0.1 °C. To be kept straight, the fiber was stretched with a tiny tension.

## Additional Information

**How to cite this article**: Zhang, H. *et al*. Highly sensitive strain sensor based on helical structure combined with Mach-Zehnder interferometer in multicore fiber. *Sci. Rep.* **7**, 46633; doi: 10.1038/srep46633 (2017).

**Publisher's note:** Springer Nature remains neutral with regard to jurisdictional claims in published maps and institutional affiliations.

## Supplementary Material

Supplementary Information
