# Supplementary material for: Highly sensitive strain sensor based on helical structure combined with Mach-Zehnder interferometer in multicore fiber
Source: Sci Rep. 2017 Apr 18;7:46633. doi: 10.1038/srep46633 (PMC5394535; doi:10.1038/srep46633)
Supplement: Supplementary Information [file srep46633-s1.pdf]

Supplementary Information

Highly sensitive strain sensor based on helical structure combined with Mach-Zehnder interferometer in multicore fiber

Hailiang Zhang[1,2], Zhifang Wu[1,2*], Perry Ping Shum[1,2*], Xuan Quyen Dinh[1,3], Chun Wah Low[1,2], Zhilin Xu[1,2], Ruoxu Wang[4], Xuguang Shao[2], Songnian Fu[4], Weijun Tong[5] & Ming Tang[4*]

[1]CINTRA CNRS/NTU/Thales, UMI 3288, 50 Nanyang Drive, Singapore 637553

[2]COFT, School of EEE, Nanyang Technological University, 50 Nanyang Avenue, Singapore 639798

[3]Thales Solutions Asia Pte Ltd, R&T, 28 Changi North Rise, Singapore 498755

[4]National Engineering Laboratory for Next Generation Internet Access System, School of Optical and Electronic Information, Huazhong University of Science and Technology, Wuhan 430074, China

[5]Yangtze Optical Fibre and Cable Company Ltd (YOFC), 4# Guanshan Er Road, Wuhan 430073, China

**Corresponding authors: zfwu@ntu.edu.sg; epshum@ntu.edu.sg; tangming@mail.hust.edu.cn*

If the lengths of the MMFs are too long, interference will be generated in the MMF sections, which will affect the sensor performance. When the length of the MMF is short enough, the accumulated phase differences of the guided modes propagating along the MMF can be neglected. To investigate the influence of the MMF length, a segment of MMF with three different lengths was spliced between two SMFs, and the transmission spectral were recorded, respectively. For easy fabrication, the lengths of the MMF were selected to be 1 mm, 2 mm and 3 mm. The spectra of the SMF-MMF-SMF structures are illustrated in Fig. S1. As can be seen, when the length was 1 mm, almost no interference was generated, hence the phase difference of its guided modes can be neglected. The interference appeared when the length was 2mm, and more obvious interference was formed for the length of 3 mm. Thus, for the proposed sensor, as the MMF length was 1 mm, the MMF almost did not generate interference spectrum by itself.

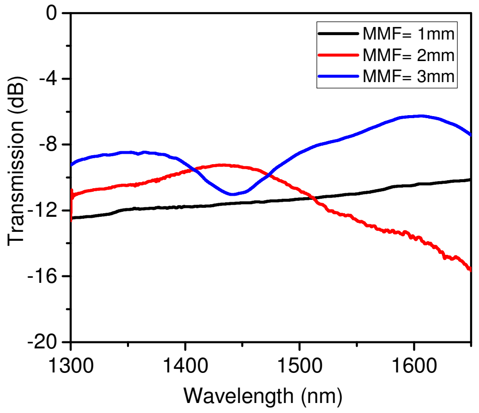

Figure S1. Transmission spectra of the SMF-MMF-SMF structures

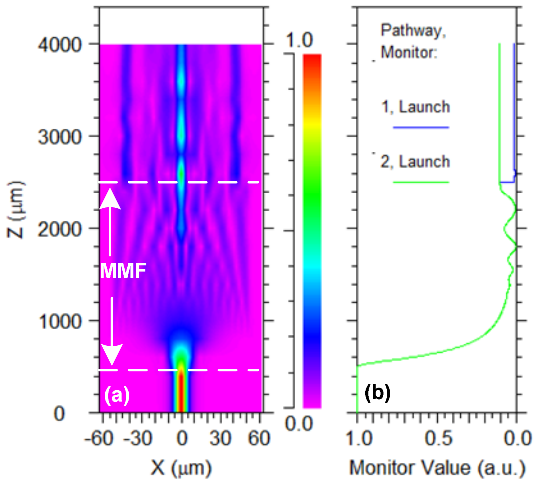

Figure S2. (a) Simulated light propagation and (b) normalized propagating power along the SMF-MMF-MCF (without HSs) structure.

To further investigate the influence of the MMF length on the coupling efficiency, beam propagation method (BPM) was utilized to simulate the light propagation in a SMF-MMF-MCF structure. Since the interference appeared when the MMF length was 2mm, to avoid the MMF generating interference by it itself, the MMF length should be less than 2 mm. For the simulation, the length of the SMF, MMF and MCF were 500 μm, 2000 μm and

2500 μm, respectively. The other values for the simulation were the same with those in the main article. Figure S2(a) shows the simulated electric field intensity distribution along the SMF-MMF-MCF structure at the wavelength of 1550 nm. Figure S2(b) shows the stimulated normalized propagating powers along the SMF-MMF-MCF structure for pathway 1 and pathway 2. Pathway 1 was from the SMF core to the MMF core, and then to one of the outer cores of the MCF. Pathway 2 was from the SMF core to the MMF core, and then to the center core of the MCF. As shown in Fig. S2(b), the normalized propagating powers decreased dramatically along the first 500 μm section of the MMF. During the section from 500 μm to 2000 μm of the MMF, the powers changed slightly. For easy fabrication of the sensor, the lengths of the MMFs were chosen to be 1 mm. Moreover, larger or smaller insertion loss caused by the MMF will not obviously affect the proposed sensor performance.

Since the FSR is approximately to the wavelength spacing between the adjacent interference dips or peaks, the FSR for the three different interference compositions can be approximately expressed by the following formulas, respectively:

$$\begin{aligned} FSR_1 &\approx \lambda_{1,m} - \lambda_{1,m+1} \\ &\approx \frac{\lambda_0^2}{\left(n_{eff}^{ce} - n_{eff}^{cl}\right)\left(L - L_H\right) + \left(n_{eff}^{ceH} - n_{eff}^{clH}\right)L_H} \\ &\approx \frac{\lambda_0^2}{\left(n_{eff}^{ce} - n_{eff}^{cl}\right)L} \end{aligned} \tag{S1}$$

$$\begin{aligned} FSR_2 &\approx \lambda_{2,m} - \lambda_{2,m+1} \\ &\approx \frac{\lambda_0^2}{\left(n_{eff}^{ou} - n_{eff}^{ce}\right)\left(L - L_H\right) + \left(n_{eff}^{ouH} L_{ouH} - n_{eff}^{ceH} L_H\right)} \\ &\approx \frac{\lambda_0^2}{\left(n_{eff}^{ou} - n_{eff}^{ce}\right)L + n_{eff}^{ou}(L_{ouH} - L_H)} \end{aligned} \tag{S2}$$

$$\begin{aligned} FSR_3 &\approx \lambda_{3,m} - \lambda_{3,m+1} \\ &\approx \frac{\lambda_0^2}{\left(n_{eff}^{ou} - n_{eff}^{cl}\right)\left(L - L_H\right) + \left(n_{eff}^{ouH} L_{ouH} - n_{eff}^{clH} L_H\right)} \\ &\approx \frac{\lambda_0^2}{\left(n_{eff}^{ou} - n_{eff}^{cl}\right)L + n_{eff}^{ou}(L_{ouH} - L_H)} \end{aligned} \tag{S3}$$

where λ_0 is the center wavelength. According to Eq. (S1), the helical length L_H does not affect the FSR of the interference generated from the center core mode and the cladding mode. According to Eq. (S2) and Eq. (S3), the FSRs for the other two types of interferences increase with reduction of $(L_{ouH,\varepsilon} - L_H)$. As show in Fig. 3 in the main article, $(L_{ouH,\varepsilon} - L_H)$ decreases nonlinearly with increasing L_H .Thus longer L_H can result in larger FSRs for the interferences generated by the outer core mode interfering with the center core mode or the cladding mode. Actually, for the fabrication of the helical structures mentioned in this work, as the heating zone of the CO_2 laser cannot be changed, the L_H is almost a constant.
